# Mechanistic role of glycolytic pathways in the tumor microenvironment in driving chemoresistance

**DOI:** 10.3389/fcell.2026.1883537

**Published:** 2026-07-22

**Authors:** Zhongyi Tan, Lai Wen, Hewen Guan, Nabuqi Bao, Zijing Wu, Xu Ji, Lin Lin

**Affiliations:** 1 First Affiliated Hospital of Dalian Medical University, Dalian, China; 2 Liaoning University of Traditional Chinese Medicine, Shenyang, China

**Keywords:** chemoresistance, glycolytic pathways, metabolic reprogramming, tumor microenvironment, lactate

## Abstract

Tumor glycolysis reprogramming, characterized by the “Warburg effect,” has emerged as a critical hallmark of cancer progression and therapeutic resistance. Increasing evidence indicates that enhanced glycolytic activity not only supports rapid tumor growth by sustaining ATP production and biosynthetic demands, but also profoundly contributes to the development of chemoresistance. In resistant tumors, glycolysis-driven metabolic adaptation promotes energy homeostasis, maintains redox balance, enhances DNA damage repair, suppresses apoptosis, and supports cancer stemness, thereby reducing the cytotoxic efficacy of chemotherapeutic agents. Moreover, aberrant glycolytic metabolism extensively remodels the tumor microenvironment (TME) through lactate accumulation, extracellular acidification, hypoxia maintenance, immune suppression, and metabolic crosstalk with stromal cells, collectively facilitating tumor survival and therapeutic tolerance. Importantly, targeting glycolytic pathways has shown promising potential in restoring chemosensitivity and enhancing the efficacy of conventional chemotherapy in multiple malignancies. In this review, we systematically summarize the role of glycolytic reprogramming in maintaining resistant tumor cell metabolic homeostasis, regulating the chemoresistant TME, and driving molecular mechanisms underlying chemotherapy resistance. We further discuss current therapeutic strategies targeting glycolysis and their potential clinical applications for overcoming chemoresistance. A deeper understanding of glycolysis-mediated metabolic plasticity may provide novel insights into precision metabolic intervention and combination therapy in cancer treatment.

## 1 Introduction

Although chemotherapy remains a cornerstone of treatment for malignant tumors, the emergence of chemoresistance continues to limit its clinical efficacy and contributes to tumor recurrence, metastasis, and poor prognosis. Chemoresistance was traditionally attributed mainly to tumor cell–intrinsic alterations, such as genetic mutations, enhanced drug efflux, activation of DNA damage repair pathways, and resistance to apoptosis (Mansoori et al., 2017; Bukowski et al., 2020). However, accumulating evidence indicates that chemoresistance is also profoundly shaped by the tumor microenvironment (TME) (Zhao et al., 2024), where hypoxia (Chen et al., 2023), acidosis (Pillai et al., 2019), immunosuppression (Liu et al., 2024), and extracellular matrix remodeling (Feng et al., 2022; Masuda, 2025) collectively enhance tumor cell adaptation to chemotherapeutic stress. Among these factors, glycolytic metabolic reprogramming, classically represented by the Warburg effect, has emerged as one of the core mechanisms linking tumor metabolism with TME remodeling and therapeutic resistance. Beyond meeting the bioenergetic and biosynthetic demands of rapidly proliferating tumor cells, enhanced glycolysis promotes lactate accumulation, extracellular acidification, immune suppression, and the maintenance of cancer stem–like properties, thereby establishing a microenvironment that favors chemoresistance (Pérez-Tomás and Pérez-Guillén, 2020; Chen et al., 2020; Liu et al., 2021).

Recent advances have further expanded our understanding of glycolysis-driven chemoresistance. Lactate is now recognized not merely as an end product of glycolysis, but as a bioactive metabolite that regulates tumor progression, immune evasion, and therapeutic response. In recent years, an increasing body of evidence has demonstrated that lactate in a variety of tumor types not only participates in metabolic reprogramming, but also functions as a key metabolic signaling molecule. It regulates cellular metabolic homeostasis, epigenetic modifications, and immune regulatory processes within the tumor microenvironment, thereby enhancing the adaptive survival capacity of tumor cells and ultimately driving the initiation and progression of chemoresistance (Jin et al., 2025; Xiang et al., 2026; Li et al., 2026; Dong et al., 2022). Notably, a 2024 mechanistic study published in Nature demonstrated that lactate promotes non-histone lactylation of the DNA repair protein NBS1, which enhances homologous recombination repair and increases cancer cell tolerance to chemotherapy. Inhibition of lactate production attenuated this process and improved chemosensitivity Lactate helps cancer cells resist (Chen H. et al., 2024). In parallel, growing evidence has shifted attention toward a TME-integrated view of glycolysis-driven chemoresistance, in which metabolic crosstalk among tumor cells, immune cells, and stromal cells coordinates an adaptive network that protects tumor cells from chemotherapy-induced damage (Zhao et al., 2024; Chen S. et al., 2024). These findings indicate that glycolytic reprogramming is not only a hallmark of tumor metabolic dysregulation, but also a critical nexus connecting metabolic remodeling, TME adaptation, epigenetic regulation, and chemotherapeutic response. This review emphasizes glycolysis as a systemic metabolic hub that integrates tumor cell survival programs with TME remodeling and therapy-induced adaptive responses. We systematically summarize how glycolytic pathways contribute to chemoresistance from three interconnected perspectives: maintenance of tumor cell energy and redox homeostasis, glycolysis-mediated remodeling of the immune and stromal microenvironment, and resistance mechanisms associated with different classes of chemotherapeutic agents. Particular attention is given to recent breakthroughs in lactate signaling, lactylation, metabolic crosstalk, and immunometabolic regulation. Furthermore, in light of recent advances in lactate signaling, metabolic crosstalk, and immunometabolic regulation, we discuss the potential clinical value of targeting the glycolysis–TME axis for overcoming chemoresistance. Collectively, this review aims to provide new mechanistic insights and a theoretical basis for the development of precision oncology and metabolism-targeted therapeutic strategies.

## 2 Metabolic reprogramming of glycolysis in diverse chemoresistant tumors

**FIGURE 1:**
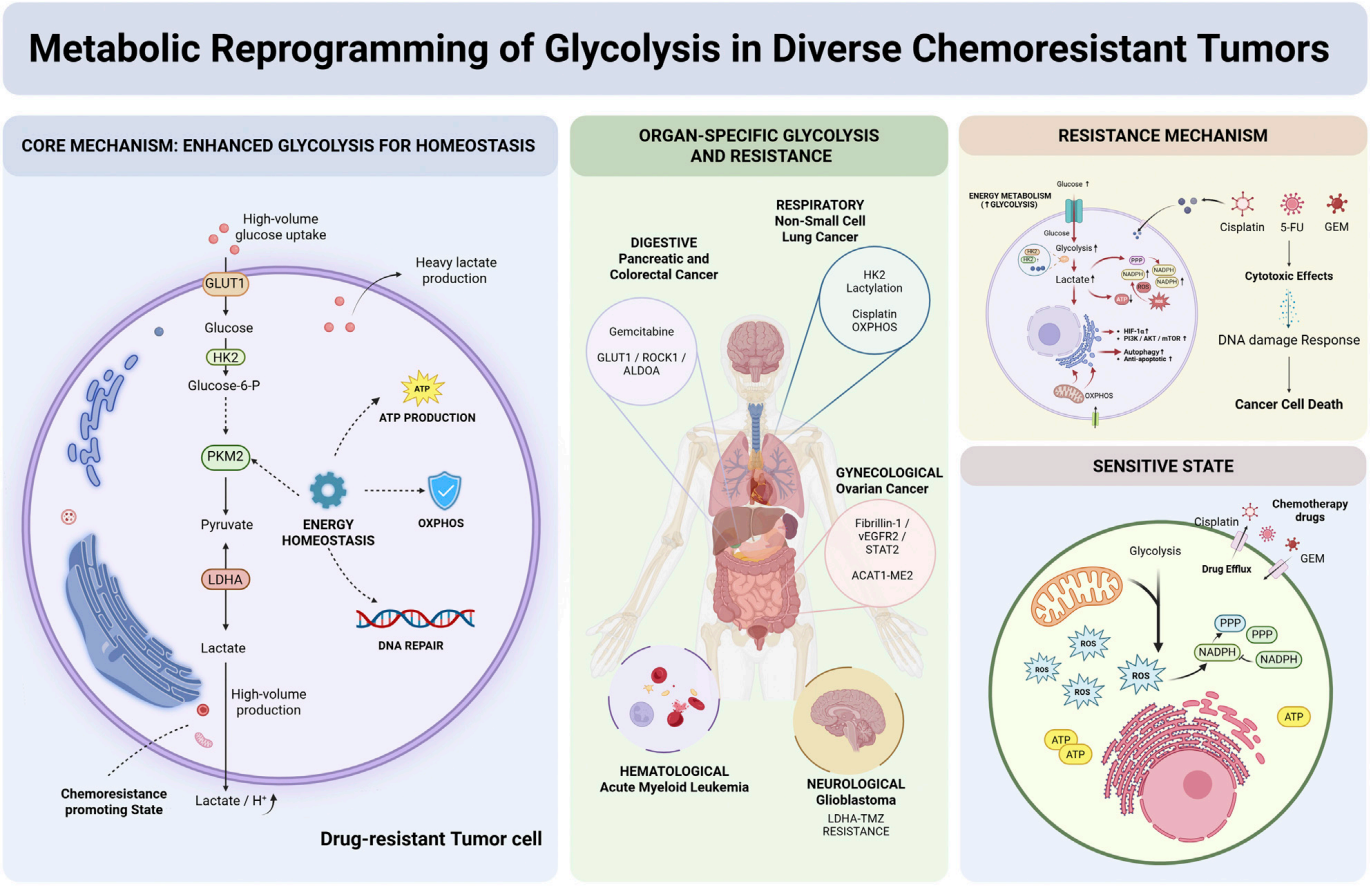
Metabolic reprogramming of glycolysis in diverse chemoresistant tumors. Figure is created by using BioRender.

To survive under continuous proliferative demands and chemotherapeutic stress, tumor cells rely on highly active metabolic programs to maintain energy homeostasis. Emerging evidence indicates that aberrant activation of glycolytic pathways not only enables rapid ATP generation in chemoresistant tumor cells, but also facilitates redox balance, biosynthetic activity, and cellular adaptation to stress, thereby promoting resistance to chemotherapy. Consistently, dysregulation of glycolysis-associated enzymes and metabolic pathways has been widely implicated in chemoresistance across multiple cancer types (Figure 1).

### 2.1 Digestive system tumors

Glycolytic reprogramming has been widely recognized as a key mechanism supporting the survival of chemoresistant cells in digestive system tumors, including pancreatic cancer, colorectal cancer, and gastric cancer. Among these malignancies, pancreatic cancer exhibits a particularly strong dependence on glycolysis due to its chronically hypoxic and nutrient-deprived microenvironment. The glycolytic pathway is one of the key mechanisms underlying gemcitabine resistance in pancreatic cancer. Targets such as SETD2, ROCK1, and ALDOA may serve as critical nodes for inhibiting tumor proliferation and significantly enhancing the therapeutic efficacy of gemcitabine (Shen et al., 2022; Pang et al., 2024; Han et al., 2023). This metabolic shift enhances glucose uptake and lactate production, thereby sustaining ATP generation and mitigating chemotherapy-induced oxidative stress, ultimately reducing apoptotic sensitivity. KRAS is one of the most important driver genes in gastrointestinal tumors, and acquired resistance remains one of the major challenges in its clinical translation. KRAS inhibitor resistance is closely associated with glycolytic reprogramming, whereby resistant tumor cells rely on glycolysis for energy production and biosynthesis, thereby escaping KRAS-targeted therapeutic pressure (Shi et al., 2025). Similarly, in colorectal cancer, enhanced glycolysis has been closely associated with resistance to 5-fluorouracil (5-FU) (Dong et al., 2022; Xin et al., 2025). The PI3K/AKT/mTOR signaling pathway drives and hyperactivates glycolytic metabolism in tumor cells, conferring the energy and metabolic advantages required for survival under drug pressure, thereby inducing and exacerbating tumor drug resistance (Dong et al., 2022; Zhong et al., 2022).

### 2.2 Respiratory system tumors

Aberrant glycolytic reprogramming is increasingly recognized as a fundamental metabolic mechanism driving resistance to cisplatin and paclitaxel in non-small cell lung cancer (NSCLC). Cisplatin-resistant NSCLC cells display enhanced glycolytic activity and increased lactylation of HK2 (Zeng et al., 2026). Studies have demonstrated that cisplatin-resistant NSCLC cell lines exhibit enhanced oxidative metabolic capacity, and inhibition of oxidative phosphorylation can effectively reverse drug resistance (Cruz-Bermúdez et al., 2019). Increased glycolytic flux not only provides sufficient ATP to support the activity of ATP-dependent drug efflux transporters, but also facilitates NADPH production, thereby enhancing reactive oxygen species (ROS) detoxification and reducing chemotherapy-induced oxidative stress (Liberti and Locasale, 2016; Marcucci and Rumio, 2021; Cunha et al., 2023). In the hypoxic tumor microenvironment, HIF-1α further promotes the transcription of glycolysis-related genes, enabling tumor cells to maintain metabolic homeostasis and adapt to both hypoxic and chemotherapeutic stress, ultimately promoting chemoresistance (Xia et al., 2018; Sowa et al., 2017).

### 2.3 Gynecologic tumors

Enhanced glycolytic metabolism has emerged as a critical contributor to chemoresistance in gynecologic tumors, particularly ovarian cancer and cervical cancer. The Fibrillin-1/VEGFR2/STAT2 signaling axis promotes chemoresistance in ovarian cancer organoids and cells by modulating glycolysis and angiogenesis (Wang et al., 2022). ACAT1-mediated acetylation of ME2 at lysine 156 promotes glutamine metabolism to produce lactate, thereby enhancing acquired chemotherapy resistance in ovarian cancer cells following long-term treatment (Zheng et al., 2025). Moreover, glycolysis-derived metabolites can promote mitochondrial metabolic adaptation and activate anti-apoptotic signaling pathways, thereby reducing chemotherapy-induced cell death (Desbats et al., 2020). This metabolic reprogramming can further enhance autophagic activity, enabling tumor cells to maintain metabolic homeostasis and survive under chemotherapeutic stress, ultimately promoting chemoresistance (Das et al., 2018).

### 2.4 Hematologic malignancies

Glycolytic metabolism has emerged as a critical energy source supporting chemoresistance in hematologic malignancies, including acute myeloid leukemia (AML) and multiple myeloma. In drug-resistant leukemia cell lines, HK activity and glycolytic capacity are enhanced, and inhibition of glycolysis is sufficient to reduce glucose consumption and thereby reverse doxorubicin resistance (Yang et al., 2024). Furthermore, leukemia stem cells exhibit pronounced metabolic plasticity and can dynamically switch between glycolysis and OXPHOS, indicating that therapy-resistant hematologic malignancies may contain metabolically heterogeneous cell populations with distinct energy dependencies. This adaptive metabolic flexibility enables them to better tolerate chemotherapeutic stress and contributes to the persistence of treatment resistance (Kreitz et al., 2019; Chen et al., 2021).

### 2.5 Nervous system tumors

A strong reliance on glycolytic metabolism is hallmark features of glioblastoma (GBM). Targeting metabolic reprogramming in glioblastoma can overcome chemoresistance in GBM (Wolf et al., 2011). Koukourakis et al. (2017) demonstrated that targeting LDHA suppresses glycolytic activity in GBM cell lines and improves their sensitivity to temozolomide (TMZ). The combination of metformin and chemotherapy in GBM treatment has entered Phase II clinical trials, and its mechanism involves inducing GBM cell death and reducing tumor growth by inhibiting OXPHOS and decreasing ATP production (Sesen et al., 2015; Bi et al., 2020). Increased glycolytic flux also promotes lactate accumulation and extracellular acidification, thereby contributing to the formation of an acidic tumor microenvironment that facilitates tumor invasion and maintenance of the resistant phenotype (Reuss et al., 2021; Seyfried et al., 2022). Furthermore, glioblastoma stem-like cells exhibit substantial metabolic adaptability and can coordinately regulate glycolysis and lipid metabolism to maintain energy homeostasis (D’Aprile et al., 2025).

Taken together, the tumor type–specific evidence summarized above indicates that glycolysis-driven chemoresistance converges on several shared adaptive mechanisms. Across diverse cancer types, enhanced glycolytic flux sustains ATP production, supports ATP-dependent processes such as drug efflux and DNA damage repair, cooperates with the pentose phosphate pathway to maintain and promotes lactate accumulation and extracellular acidification, thereby facilitating tumor cell adaptation under chemotherapeutic stress. Glycolytic reprogramming is also frequently associated with activation of survival signaling, maintenance of cancer stem–like properties, autophagy, and enhanced stress tolerance. However, these shared mechanisms do not indicate that all chemoresistant tumors rely on glycolysis to the same extent. For example, pancreatic cancer cells in chronically hypoxic and nutrient-deprived microenvironments often exhibit strong glycolytic dependence, whereas stem-like resistant populations in colorectal cancer, leukemia, and glioblastoma may display greater metabolic plasticity. These cells can maintain survival by coordinating glycolysis with OXPHOS, lipid metabolism, or PPP-dependent redox regulation, thereby adapting to chemotherapy-induced energy stress, oxidative damage, and nutrient limitation. This metabolic heterogeneity suggests that glycolysis-driven chemoresistance should be understood as a context-dependent adaptive program rather than a uniform metabolic phenotype across all tumor types.

## 3 Aberrant glycolytic pathways remodel the tumor microenvironment and facilitate chemoresistance

**FIGURE 2:**
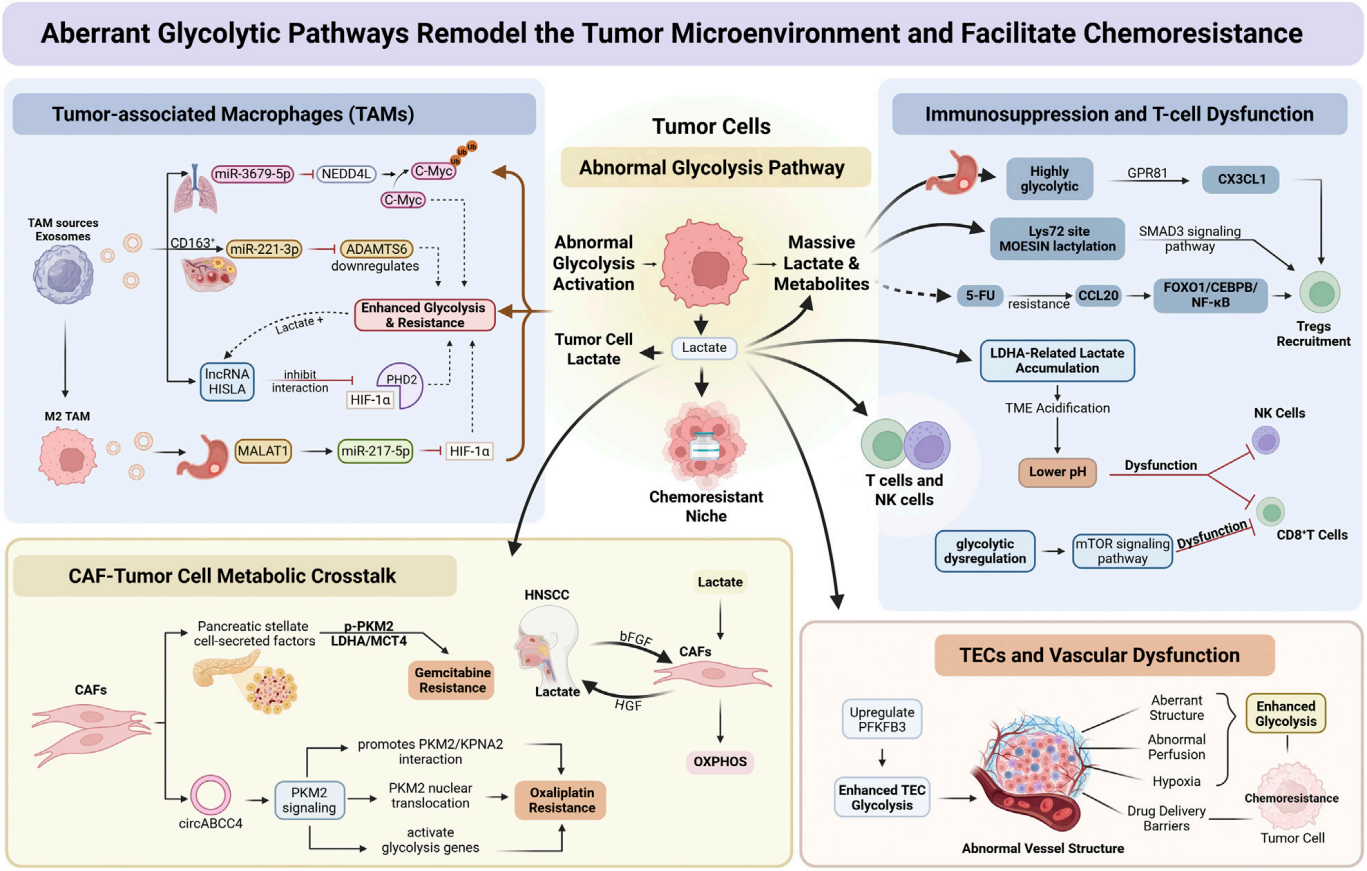
Aberrant glycolytic pathways remodel the tumor microenvironment and facilitate chemoresistance. Figure is created by using BioRender.

Beyond its role in tumor cell–intrinsic metabolic reprogramming, aberrant activation of glycolytic pathways can profoundly remodel TME, thereby facilitating the formation of a chemoresistant niche that supports tumor survival and therapeutic tolerance. Enhanced glycolytic flux leads to excessive accumulation of lactate and other metabolic intermediates, which can induce extracellular acidification, regulate immune cell function, and promote metabolic crosstalk among multiple cellular components within the TME. As a consequence, glycolysis-driven metabolic alterations broadly influence tumor-associated macrophages (TAMs), regulatory T cells (Tregs), myeloid-derived suppressor cells (MDSCs), and cancer-associated fibroblasts (CAFs), ultimately contributing to immunosuppression, stromal remodeling, and maintenance of cancer stem–like phenotypes. Taken together, these findings suggest that glycolytic reprogramming is not merely a hallmark of malignant metabolic dysregulation, but also a central mechanism underlying microenvironment-mediated adaptive resistance to chemotherapy (Figure 2).

### 3.1 TAMs

TAMs have emerged as critical regulators of chemoresistance by promoting aberrant glycolytic reprogramming in tumor cells through multiple mechanisms. Through the extracellular vesicle (EV)-mediated transfer of HISLA—a myeloid-specific lncRNA—TAMs augment aerobic glycolysis and apoptotic evasion in breast cancer cells, which is achieved as HISLA impedes PHD2 from binding to HIF-1α, thus halting its hydroxylation and degradation (Chen et al., 2019). Similarly, by impeding NEDD4L to prevent c-Myc degradation, macrophage-derived exosomes drive both aerobic glycolysis and resistance to chemotherapy in lung cancer (Wang et al., 2020). TAM-derived exosomes of M2 phenotype upregulate HIF-1α protein expression via MALAT1, jointly promoting aerobic glycolysis in gastric cancer cells. Targeted inhibition of MALAT1 can suppress M2-polarized macrophage–mediated gastric cancer progression and enhance chemotherapy efficacy (Wang et al., 2024). Beyond these directly demonstrated glycolysis-dependent mechanisms, TAM-derived exosomes may also engage signaling pathways with potential metabolic relevance. In epithelial ovarian cancer, CD163^+^ TAM-derived exosomal miR-221–3p downregulates ADAMTS6 and activates AKT signaling, promoting proliferation, adhesion, migration, and drug resistance in EOC cells; however, its association with glycolytic reprogramming remains unclear (Zhang X. et al., 2022).

### 3.2 Tregs

Tregs are an important component of the immunosuppressive microenvironment shaped by tumor glycolysis. In highly glycolytic gastric cancer, lactate/GPR81 signaling modulates CX3CL1 and promotes Treg recruitment into the tumor microenvironment (Su et al., 2024). Lactate can also induce MOESIN lactylation at the Lys72 site, thereby enhancing the interaction between MOESIN and TGF, activating the SMAD3 signaling pathway, and subsequently regulating Treg cells (Gu et al., 2022). In colorectal cancer, 5-fluorouracil-treated tumor cells recruit Tregs through the FOXO1/CEBPB/NF-κB/CCL20 axis, thereby promoting chemoresistance (Wang et al., 2019). Although these findings collectively implicate Tregs in glycolysis-associated immunosuppressive remodeling and chemotherapy resistance, direct evidence that lactate-driven Treg reprogramming causally mediates chemoresistance remains limited.

### 3.3 CD8^+^ T cells/NK cells

CD8^+^ T cells and natural killer (NK) cells within highly glycolytic tumors are frequently exposed to glucose deprivation, lactate accumulation, and extracellular acidification, all of which can compromise their metabolic fitness and antitumor effector functions. In melanoma, excessive lactate accumulation driven by LDHA promotes extracellular acidification and reduces intratumoral pH, which markedly impairs the proliferation, cytokine secretion, and cytotoxic functions of CD8^+^T cells and NK cells, ultimately facilitating tumor immune evasion (Brand et al., 2016). In addition, glycolytic dysregulation may contribute to functional impairment of CD8^+^T cells through modulation of the mTOR signaling pathway, accompanied by reduced IFN-γ production (Cao et al., 2023). Furthermore, aerobic glycolysis represents a hallmark metabolic feature of NK cell activation and demonstrated that LDHA-deficient NK cells exhibit antitumor effector functions (Sheppard et al., 2021).

### 3.4 MDSCs

Glycolysis reshapes the MDSC-mediated immunosuppressive microenvironment, creating favorable conditions for tumor survival and treatment tolerance. In triple-negative breast cancer (TNBC), tumor glycolytic activity has been shown to regulate MDSC function through a coordinated molecular network involving the AMPK–ULK1 axis, autophagy, and CEBPB signaling, thereby maintaining tumor-associated immunosuppression (Li et al., 2018). In pancreatic ductal adenocarcinoma (PDAC), targeting tumor-derived lactate may help alleviate radioresistance by modulating HIF-1α signaling in MDSCs (Yang et al., 2020).

### 3.5 CAFs

Within the drug-resistant tumor microenvironment, CAFs promote chemoresistance by inducing glycolytic reprogramming through paracrine signaling, metabolite exchange, and exosome-mediated communication. In head and neck squamous cell carcinoma, CAF-derived HGF has been reported to enhance glycolysis and increase extracellular lactate production in tumor cells, while tumor-derived basic fibroblast growth factor in turn sustains CAF activation, thereby establishing a reciprocal metabolic crosstalk loop between CAFs and cancer cells (Kumar et al., 2018). Under glucose-limited conditions, CAFs can utilize lactate released from highly glycolytic cancer cells as an alternative energy source. Additionally, lPDAC cells enhance LDHA-driven glycolysis and lactate production, leading to glucose deprivation in the TME; CAFs utilize tumor-derived lactate for metabolic adaptation and induce IL-6 expression, thereby promoting an immunosuppressive microenvironment and tumor progression (Kitamura et al., 2023). Pancreatic stellate cell-secreted factors enhance LDHA- and MCT4-associated glycolysis in pancreatic cancer cells, thereby reducing gemcitabine sensitivity (Amrutkar et al., 2023). Similarly, circABCC4 promotes the interaction between PKM2 and KPNA2, facilitating PKM2 nuclear translocation in CAFs and subsequently activating the transcription of glycolysis-related genes, ultimately driving oxaliplatin resistance in PDAC (He et al., 2025).

### 3.6 Tumor endothelial cells

Abnormal glycolytic activity in tumor endothelial cells (TECs) contributes to the formation of dysfunctional tumor vasculature, characterized by disorganized vessel architecture, aberrant perfusion, and increased permeability, ultimately leading to hypoxia and impaired drug delivery within tumor tissues. These alterations can indirectly enhance glycolysis in tumor cells and promote chemoresistance. Mechanistically, PFKFB3-driven endothelial glycolysis promotes abnormal tumor vasculature and impaired perfusion, whereas partial PFKFB3 inhibition normalizes tumor vessels, improves chemotherapeutic drug delivery, and enhances treatment efficacy (Cantelmo et al., 2016; Matsumo et al., 2021).

Collectively, the evidence discussed above suggests that glycolytic reprogramming in the chemoresistant TME functions as an interconnected metabolic communication network rather than an isolated alteration within a single cell population. In this network, lactate acts as a central mediator linking tumor cells with immune, stromal, and endothelial compartments. Lactate released by highly glycolytic tumor cells can acidify the extracellular microenvironment and suppress the cytotoxic activity of CD8^+^ T cells and NK cells, while also contributing to Treg recruitment, MDSC-associated HIF-1α signaling, and TAM-related feedback regulation. In the stromal compartment, tumor-derived lactate can be utilized by CAFs as an alternative energy source and stimulate IL-6 production, thereby linking tumor glycolysis with stromal activation and immune suppression. Conversely, TAMs and CAFs can further reinforce tumor glycolysis and chemoresistance through exosomal non-coding RNAs, cytokine secretion, and paracrine signaling pathways involving HIF-1α, and PKM2. In addition, abnormal glycolysis in TECs aggravates vascular dysfunction, hypoxia, and insufficient drug delivery, creating conditions that further increase tumor glycolytic dependence. Thus, lactate-mediated metabolic crosstalk coordinates reciprocal communication among tumor cells, immune cells, stromal cells, and endothelial cells, ultimately connecting immune suppression, stromal remodeling, vascular abnormality, and chemotherapy resistance.

## 4 Glycolytic pathways drive chemotherapy resistance through multiple molecular mechanisms

Increasing evidence suggests that, beyond driving metabolic reprogramming in tumor cells, aberrant activation of glycolytic pathways also directly promotes resistance to multiple chemotherapeutic agents. Through glycolytic reprogramming, tumors can decrease their sensitivity to chemotherapeutic agents by maintaining ATP production and redox balance, enhancing DNA damage repair, suppressing apoptosis, and preserving cancer stem–like characteristics. Meanwhile, glycolysis-associated changes, such as lactate accumulation, sustained hypoxia, and metabolic crosstalk, further promote adaptation of the tumor microenvironment and facilitate immune evasion. Importantly, different chemotherapeutic agents exhibit distinct glycolysis-dependent mechanisms of resistance. Therefore, a systematic understanding of the molecular mechanisms by which glycolytic pathways mediate resistance to specific chemotherapy drugs may facilitate the development of novel metabolism-targeted combination therapies.

### 4.1 Cisplatin

Aberrant activation of glycolysis can reduce the sensitivity of tumor cells to platinum-based agents such as cisplatin by promoting metabolic adaptation, maintaining redox balance, and enhancing DNA damage repair, ultimately leading to chemoresistance. Following intracellular activation, platinum-based agents such as cisplatin primarily exert their cytotoxic effects by inducing intra- and interstrand DNA crosslinks, which lead to activation of DNA damage response (DDR) pathways. In addition to DNA damage, these agents can also induce mitochondrial dysfunction and excessive ROS accumulation, further amplifying cellular damage and triggering mitochondria-dependent apoptosis (Dasari and Tchounwou, 2014). In bladder cancer cells and clear cell-renal cell carcinoma, increased PPP activity promotes NADPH production, which helps neutralize cisplatin-induced ROS (Gong et al., 2025; Lucarelli et al., 2015). In addition, the detachment of hexokinase II from the mitochondria markedly potentiates the onset of caspase-2 induced mitochondrial damage, thus resulting in a synergistic induction of cisplatin induced cytotoxicitys (Shulga et al., 2009).

### 4.2 Gemcitabine

Glycolytic reprogramming allows tumor cells to restore their ability to sustain DNA replication, survival, and repair, thereby overcoming gemcitabine-induced replication stress and apoptosis. Following intracellular activation, gemcitabine is a cancer drug of the anti-metabolite class. It is a deoxycytidine analog that interferes with DNA synthesis by incorporating into elongating DNA, and indirectly interferes with DNA replication by inhibiting the nucleoside salvage pathway (Alvarellos et al., 2014; Zhang et al., 2025). Increased glycolytic flux promotes glucose addiction and enhances pyrimidine biosynthesis, thereby elevating intracellular dCTP levels that competitively reduce the effective activity of gemcitabine (Shukla et al., 2017). Stabilization of HIF-1α regulated by MUC1 mediates this metabolic shift. Targeting HIF-1α or *de novo* pyrimidine biosynthesis in conjunction with gemcitabine substantially reduces tumor burden (Shukla et al., 2017).

### 4.3 Paclitaxel

Glycolytic reprogramming promotes paclitaxel resistance by enhancing tumor-cell survival and suppressing drug-induced apoptosis. PDK2 expression is upregulated in paclitaxel-resistant lung cancer cells (Sun et al., 2017). Knockdown of PDK2 enhances the sensitivity of resistant lung cancer cells to paclitaxel by reducing glycolytic activity, rather than by enhancing oxidative phosphorylation (Sun et al., 2017). Additionally, co-treatment with paclitaxel and dichloroacetate—a targeted PDK2 inhibitor—synergistically attenuates the survival of lung cancer cells exhibiting paclitaxel resistance (Sun et al., 2017). In addition, Xu et al. (2023) reported that the E3 ubiquitin ligase TRAF6 regulates glycolysis and promotes paclitaxel chemoresistance in triple-negative breast cancer by directly binding to PKM2.

### 4.4 5-FU

As a nucleotide analog, 5-FU enters tumor cells and disrupts DNA and RNA synthesis and function, ultimately inducing tumor cell death (Noordhuis et al., 2004). However, despite its ability to impair DNA synthesis, cancer cells can adapt to 5-FU-induced stress through metabolic reprogramming, including enhanced reliance on OXPHOS and remodeling of pentose phosphate pathway activity (Denise et al., 2015).

## 5 Targeting glycolysis to overcome chemoresistance

Notably, an increasing number of studies have highlighted the therapeutic potential of combining glycolysis-targeted strategies with conventional chemotherapy to overcome tumor chemoresistance. In colorectal cancer, inhibition of PKM2-driven glycolysis has been shown to restore sensitivity to 5-FU (Wu et al., 2022). In pancreatic cancer cell lines, co-treatment with the glycolytic inhibitor 2-deoxy-D-glucose (2-DG) markedly enhances the antitumor efficacy of both 5-FU and gemcitabine (Cheng et al., 2014; Dai et al., 2020). Likewise, 2-DG treatment has been reported to re-sensitize ovarian cancer organoids to cisplatin (Chen et al., 2026). Mechanistically, HK2 promotes cisplatin resistance by enhancing drug-induced ERK-mediated autophagy (Zhang et al., 2018). Meanwhile, studies have demonstrated that inhibition of PFKFB3 can enhance the therapeutic response of drug-resistant tumor cells to paclitaxel, carboplatin (CBPt), and cisplatin (Cis) by suppressing metabolic adaptation and reducing lactate production (Xiao et al., 2021; Lu et al., 2021). Taken together, these findings suggest that targeting glycolysis not only disrupts the metabolic plasticity of resistant tumor cells, but also remodels the tumor microenvironment and resistance-associated signaling networks, providing a promising therapeutic strategy for overcoming chemoresistance.

Although these findings support the potential of glycolysis-targeted chemosensitization, most current evidence remains preclinical. Several metabolism-targeted agents, including 2-DG (Mohanti et al., 1996), lonidamine (Dogliotti et al., 1996), dichloroacetate (Dunbar et al., 2014), and the MCT1 inhibitor AZD3965 (Halford et al., 2023), have entered clinical evaluation, either alone or in combination with chemotherapy, radiotherapy, or other anticancer therapies. However, these studies were not uniformly designed to reverse established chemoresistance, and glycolysis-targeted interventions have not yet been incorporated into standard clinical practice for overcoming chemotherapy resistance. Major translational challenges include insufficient tumor selectivity, potential systemic toxicity, and compensatory metabolic adaptation involving oxidative phosphorylation, glutamine metabolism, and other alternative energy or biosynthetic pathways. In addition, systemic glycolytic inhibition may affect normal cells and certain antitumor immune-cell populations, which also rely on glycolysis to support activation, proliferation, and effector functions (Stine et al., 2022; Chelakkot et al., 2023; Zhang Y. et al., 2022). Therefore, future clinical development should focus on rational combination strategies, optimized dosing schedules, improved tumor-selective delivery, and biomarker-guided patient selection.

## 6 Conclusion

As a hallmark of cancer metabolic dysregulation, glycolytic reprogramming plays a central role in the initiation and maintenance of tumor chemoresistance. Accumulating evidence suggests that enhanced glycolysis not only fulfills the elevated metabolic demands of resistant tumor cells by continuously supplying ATP, maintaining redox homeostasis, and supporting anabolic biosynthesis, but also remodels the tumor microenvironment through lactate accumulation and extracellular acidification, thereby promoting immunosuppression, tumor progression, and therapeutic resistance. Beyond its metabolic functions, glycolysis is closely linked to multiple resistance-associated mechanisms, including drug efflux, DNA damage repair, cancer stem cell maintenance, autophagy, and anti-apoptotic signaling. Through the coordinated regulation of these processes, glycolytic reprogramming ultimately drives resistance to a broad spectrum of chemotherapeutic agents, including cisplatin, gemcitabine, paclitaxel, and 5-FU.

Notably, increasing efforts in recent years have focused on elucidating the roles of key glycolytic regulators and metabolic pathways in tumor chemoresistance. Targeting critical glycolytic enzymes, such as MALAT1, PFKFB3, and PKM2, has been shown to partially restore tumor cell sensitivity to chemotherapeutic agents and enhance the efficacy of conventional chemotherapy. These findings highlight glycolysis not only as a central biological driver of chemoresistance, but also as a promising therapeutic target for reversing drug resistance. Despite these advances, the clinical translation of glycolysis-targeted therapies remains challenging. Major obstacles include compensatory activation of alternative metabolic pathways, toxicity to normal tissues, the lack of robust, and reliable metabolic biomarkers. Looking forward, advances in technologies such as single-cell sequencing, multi-omics integration, spatial metabolomics, and liquid biopsy are expected to provide deeper insight into the dynamic mechanisms by which glycolysis promotes chemoresistance. A more comprehensive understanding of these processes may facilitate the development of more precise and effective metabolic intervention strategies, ultimately improving chemotherapy efficacy and patient prognosis.
